# Effects of aerobic, resistance, interval, and combined training on glucose metabolism in older adults: insights into type, dose, and mechanism

**DOI:** 10.3389/fphys.2025.1702669

**Published:** 2025-11-20

**Authors:** Qilong Zhang, Yifan Guo, Hengyi Zhang, Weiliang Xu, Lijun Yin

**Affiliations:** 1 School of Physical Education, Jiangsu University of Science and Technology, Zhenjiang, China; 2 School of Elderly Care Services and Management, Nanjing University of Chinese Medicine, Nanjing, China; 3 School of Sports, Shenzhen University, Shenzhen, China

**Keywords:** aging, glucose homeostasis, insulin resistance, exercise intervention, exercise modalities

## Abstract

Aging is accompanied by reduced skeletal muscle insulin sensitivity, abnormal fat redistribution, and a gradual decline in pancreatic β-cell function, leading to impaired glucose homeostasis and an increased risk of type 2 diabetes mellitus and related complications. Exercise is widely recognized as a core non-pharmacological strategy to improve glucose metabolism in older adults. This is a narrative review based on a comprehensive search of PubMed and Web of Science databases up to September 2025. We summarize current evidence on the effectiveness of different exercise modalities—including aerobic, resistance, high-intensity interval, and combined training—in ameliorating age-related glucose metabolism disorders. Evidence suggests that, in the long term, combined training yields more comprehensive improvements in insulin secretion and multiple metabolic markers compared with single-modality interventions. Particular attention is given to the intensity, frequency, and duration of exercise interventions to discuss dose–response characteristics and practical implications for older adults. Mechanistic insights indicate that exercise exerts its benefits through multiple pathways, including enhanced skeletal muscle GLUT4 expression and mitochondrial function, reduced visceral and ectopic adiposity, suppression of chronic low-grade inflammation, and improved β-cell insulin secretion. Future research should focus on large-scale, long-term clinical trials and mechanistic studies to refine exercise prescriptions, clarify dose–response relationships, and characterize the unique metabolic adaptations of the elderly population.

## 1 Introduction

The accelerating pace of population aging has made age-related metabolic diseases an urgent global public health challenge. Among these, disorders of glucose metabolism—including insulin resistance, elevated fasting glucose, impaired glucose tolerance, and type 2 diabetes (T2D)—are particularly prevalent in older adults (Amorim et al., 2022). These conditions not only exacerbate the progression of aging-related diseases such as cardiovascular disease, cognitive decline, and sarcopenia, but also substantially increase healthcare costs and mortality risk. Epidemiological studies indicate that more than one-third of older adults exhibit some degree of impaired glucose metabolism, and this proportion continues to rise (Bellary et al., 2021).

Among non-pharmacological interventions, exercise is widely recognized as a cornerstone strategy for improving glucose homeostasis, slowing the progression of diabetes, and enhancing quality of life (Bellary et al., 2021). Regular physical activity enhances insulin sensitivity and β-cell function by increasing skeletal muscle glucose uptake, improving insulin signaling pathways, augmenting mitochondrial function, and modulating fat distribution (Zhang T. et al., 2025; Zhang Q. et al., 2025). However, in older adults, the presence of multiple chronic conditions, age-related functional decline, and reduced exercise tolerance complicate the choice of optimal exercise modalities and dosing parameters. In accordance with the World Health Organization, “older adults” are defined as individuals aged ≥65 years. Aging, however, is a progressive process, characterized by gradual declines in multiple physiological functions, including insulin sensitivity, mitochondrial efficiency, muscle mass, and β-cell capacity. Consequently, physiological responses to exercise may vary substantially between individuals at the younger end of this spectrum and those at the older end (Gomez-Bruton et al., 2020; Lohne-Seiler et al., 2016).

This review compares the programs and effects of aerobic training, resistance training, high-intensity interval training (HIIT), and combined training interventions in improving age-related glucose metabolism disorders. Furthermore, it analyzes the dose–response relationships and targeted effects of different exercise modalities with respect to intensity, frequency, and duration, with the aim of providing evidence-based guidance for exercise prescription in older adults with impaired glucose metabolism.

## 2 Multi-organ mechanisms of aging-associated disorders of glucose metabolism

Normal glucose homeostasis requires proper insulin secretion from pancreatic β-cells and effective peripheral glucose utilization by insulin-sensitive tissues. It is estimated that 30% of individuals over the age of 60 are affected by T2D (Cowie et al., 2009). This age-related disruption in glucose metabolism is not caused by a single factor but results from the synergistic decline of various physiological functions across multiple organs and tissues.

### 2.1 Skeletal muscle

Skeletal muscle, being the primary target for insulin-mediated glucose uptake, plays a crucial role in the pathogenesis of insulin resistance in the elderly. Age-related alterations in the structure and metabolism of this tissue are thought to significantly contribute to this condition (Whytock and Goodpaster, 2025). The decline in muscle mass and function is a hallmark of the aging process, with muscle atrophy beginning as early as 25 years of age and accelerating thereafter, so that by the age of 80, approximately 40% of the lateral femoral muscles (thigh muscles) are lost (Consitt et al., 2019). Importantly, sarcopenia is considered detrimental to glucose uptake as it reduces the muscle mass available for insulin-stimulated glucose processing (Nishikawa et al., 2021). In addition to muscle mass loss, age-related metabolic and cellular changes in skeletal muscle are thought to play a more prominent role and have been a focal point for researchers investigating the intracellular mechanisms underlying age-related insulin resistance.

At the molecular level, the expression of glucose transporter protein 4 (GLUT4) declines with age in skeletal muscle. In human studies, GLUT4 was reduced by approximately 25% in type II fibers of the lateral femoral muscle in older (mean age: 64 years) compared to younger (mean age: 29 years) subjects, while no significant difference was observed in type I fibers (Gaster et al., 2000). This finding was further corroborated by animal studies in which glucose uptake rates in the soleus muscle of 4, 10, 22, and 42-week-old rats showed lower GLUT4 expression in older animals, with a negative correlation to age, while no such relationship was observed for GLUT1 expression (Dos Santos et al., 2012). More importantly, the ability of GLUT4 to translocate to the plasma membrane in response to insulin is impaired in the gastrocnemius muscle of aging mice (Deng et al., 2024). Furthermore, aging negatively impacts insulin-stimulated phosphorylation of AS160 at serine-588 and threonine-642, which are thought to have a significant combined effect on GLUT4 translocation (Consitt et al., 2013; Sano et al., 2003). In the elderly, skeletal muscle Akt activity is diminished at an early age during hyperinsulinemia, contributing to insulin resistance (Petersen et al., 2015). These studies suggest that age-related impairments in insulin signaling may reduce GLUT4 translocation from intracellular storage vesicles to the plasma membrane, ultimately leading to skeletal muscle insulin resistance. Additionally, intramuscular lipid accumulation and reduced mitochondrial function are also key contributors to insulin resistance (Kim et al., 2008).

### 2.2 Adipose tissue

Adipose tissue serves as an essential energy reservoir and endocrine organ, maintaining glycolipid and energy homeostasis throughout the body. It undergoes significant changes with aging, many of which contribute to metabolic dysfunction. Specifically, aging is associated with alterations in body fat distribution, marked by a decrease in subcutaneous fat and an increase in visceral fat, and this redistribution has been linked to heightened insulin resistance (Kuk et al., 2009). Mechanistically, the accumulation of visceral fat during aging leads to altered lipid metabolism, characterized by increased lipolysis and elevated free fatty acid levels, which may reduce peripheral insulin sensitivity (Guilherme et al., 2008). Moreover, lipid redistribution and chronic inflammation resulting from aging adipose tissue induce metabolic disorders, including insulin resistance, impaired glucose tolerance, and diabetes (Mi et al., 2019; Xiao et al., 2025). Elevated levels of pro-inflammatory cytokines, such as members of the IL-1 family, in dysfunctional adipose tissue may directly interfere with insulin signaling pathways (Stienstra et al., 2010; Ballak et al., 2015). Recent findings also suggest that cellular senescence in adipose tissue is linked to metabolic dysfunction, as inhibition of p53 activity in adipose tissue significantly alleviates insulin resistance (Minamino et al., 2009). Growing evidence indicates that age-related changes in adipose tissue contribute to insulin resistance in the elderly. Age-related defects in insulin signaling cascades, such as reduced insulin-stimulated tyrosine phosphorylation, are more pronounced in adipose tissue than in liver or muscle, suggesting that adipose tissue may be central to the development of insulin resistance with aging (Serrano et al., 2009). Additionally, age-related alterations in immune cells within adipose tissue, such as T-cell accumulation, may further contribute to insulin resistance (Bapat et al., 2015).

### 2.3 Pancreatic β-cells

T2D accounts for 90%–95% of all diabetes. This form encompasses individuals who generally have relative (rather than absolute) insulin deficiency and have insulin resistance (i.e., decreased biological responses to insulin) (American Diabetes Association Professional Practice Committee, 2025). Pancreatic β-cells in the islets of Langerhans maintain circulating normoglycemia within a narrow range through insulin secretion. To meet ongoing metabolic demands, β-cells exist in a dynamic state, undergoing turnover through replication, neogenesis, and apoptosis. In healthy individuals, β-cells exhibit a long lifespan. β-cell clusters are established during the first few years of life, and thereafter, β-cells age in parallel with the body (Saisho et al., 2013). Although age is a significant risk factor for T2D, the changes that occur in human pancreatic islets during aging have not been extensively studied. Lower β-cell proliferation rates have been reported with age (Dai et al., 2017; Aguayo-Mazzucato, 2020). Several studies indicate that β- and α-cell volumes are largely maintained in elderly non-diabetic individuals, despite the higher prevalence of diabetes in this population (Mizukami et al., 2014; Moin et al., 2020). However, the ability of β-cells to adapt to stress and metabolic demands may be impaired with age (Kushner, 2013). Aging is associated with a 0.7% annual decline in insulin secretion, attributed to a combination of β-cell dysfunction and increased β-cell apoptosis, with glucose-intolerant individuals experiencing a 50% reduction in β-cell mass (Szoke et al., 2008). Additionally, β-cell autoimmunity may contribute to the activation of the acute phase response in elderly diabetic patients (Dehghan et al., 2007). In genetically predisposed individuals, chronic overproduction of interleukins, C-reactive protein, and tumor necrosis factor-α may impair β-cell insulin secretion and contribute to insulin resistance (Dehghan et al., 2007). Thus, the diabetogenic effects of aging are characterized by increased insulin resistance and reduced insulin secretion. People with T2D early in the disease course may have insulin levels that appear normal or elevated, yet the failure to normalize blood glucose reflects a relative defect in glucose-stimulated insulin secretion that is insufficient to compensate for insulin resistance. Insulin resistance may improve with weight reduction, physical activity, and/or pharmacologic treatment of hyperglycemia but is seldom restored to normal (American Diabetes Association Professional Practice Committee, 2025). This dynamic process begins with insulin resistance in a prediabetic state, with β-cells compensating by increasing insulin secretion. Over time, however, β-cell insulin secretion becomes insufficient due to both the decreased capacity of β-cells to compensate for insulin resistance and further reductions in insulin sensitivity in peripheral tissues, ultimately progressing to persistent hyperglycemia, glucose intolerance, and diabetes.

## 3 Effects of different exercise modalities on glucose metabolism in the elderly

### 3.1 Aerobic exercise

Aerobic exercise is typically defined as continuous, rhythmic physical activity that predominantly engages large muscle groups and relies primarily on aerobic (oxidative phosphorylation) energy metabolism to generate ATP over sustained periods; common examples include walking, cycling, swimming, and jogging (Chamari and Padulo, 2015). A large body of evidence demonstrates that aerobic exercise is an effective strategy for improving glucose metabolism and insulin sensitivity in the elderly. Notably, its metabolic benefits can be observed across long-term, moderate-term, and even short-term interventions.

Long-duration programs (≥6 months) have consistently shown benefits. In overweight or obese older adults, both aerobic exercise and dietary weight loss improve insulin sensitivity, although through different mechanisms. Six months of aerobic exercise reduced the area under the insulin curve during the late oral glucose tolerance test (OGTT) phase (120–180 min), suggesting enhanced β-cell secretory capacity and improved tissue insulin sensitivity (Ryan et al., 2021). By contrast, dietary weight loss alone produced broader effects, including reductions in fasting glucose, glucose and insulin levels across the OGTT, and a 16% reduction in total body fat (visceral and subcutaneous), though with some muscle loss. Exercise, however, improved body composition by increasing lean mass, reducing intramuscular fat, and enhancing cardiorespiratory fitness despite minimal weight loss (Ryan et al., 2021). These findings indicate that diet and exercise confer complementary advantages: diet reduces fat load, whereas exercise preserves muscle and improves fitness. Their combination yields maximal metabolic benefits. Supporting this, another study demonstrated that dietary weight loss combined with aerobic exercise produced greater improvements in insulin sensitivity than diet alone, with a higher proportion of “high responders” (83% vs. 46%) and greater weight loss (−10.6 kg vs. −7.1 kg), strongly correlated with metabolic improvements (Brennan et al., 2020). Other evidence shows that aerobic exercise without caloric restriction still enhances peripheral insulin sensitivity by improving skeletal muscle glucose uptake and utilization. However, combining exercise with moderate energy restriction (∼500 kcal/day) amplifies the benefits, especially in fasting glucose and postprandial responses (Erickson et al., 2019).

Moderate-duration interventions (8–12 weeks) can yield benefits comparable to long-term training. For instance, 12 weeks of individualized maximal fat oxidation rate (FATmax) training in older women with T2Dreduced body fat, including visceral fat, improved insulin resistance, lowered glycemic and lipid levels, and enhanced cardiorespiratory fitness (Tan et al., 2018). Similarly, a 12-week program in prediabetic adults improved Glycated Hemoglobin (HbA1c), fasting insulin, HOMA-IR, and ambulatory glucose monitoring, while decreasing body fat and preserving lean mass (Konopka et al., 2019). However, findings are not always consistent. For example, 12 weeks of moderate-intensity walking (50%–60% VO_2_max) failed to reduce fasting glucose in older women (Li et al., 2025), and in older breast cancer survivors, benefits were limited to reductions in postprandial insulin levels (Viskochil et al., 2020). These discrepancies suggest that the most consistent effects of aerobic exercise are observed in the postprandial state (i.e., improved glucose load handling), rather than fasting measures.

Hypoxic training has also been examined. In sedentary older adults, 8 weeks of cycling under normobaric hypoxia or normoxia improved insulin sensitivity and glycemic indices in both groups, with no significant differences between them. This indicates that exercise itself is the primary determinant of metabolic improvements, while hypoxia provides no additional advantage (Chobanyan-Jürgens et al., 2019).

Even short-term aerobic exercise (≤2 weeks) can provide detectable benefits. Two weeks of cycling (interval or continuous) reduced postprandial glucose, improved systemic and adipose tissue insulin resistance, and increased VO_2_max, suggesting that total energy expenditure, rather than exercise intensity pattern, is the critical determinant of short-term improvement (Gilbertson et al., 2018). Similarly, low-intensity walking rapidly improved pancreatic β-cell function and reduced daily glucose within 2 weeks (Karstoft et al., 2017). Recent randomized controlled evidence indicates that short-term exercise can markedly decrease pancreatic ectopic fat and that improvement in β-cell function often co-occurs with reduced pancreatic fat (Heiskanen et al., 2018), suggesting that alleviation of glucotoxicity/lipotoxicity and ectopic pancreatic fat reduction may underlie early recovery of β-cell function after short interventions.

To summarize, aerobic exercise produces significant improvements in glucose metabolism across different intervention durations. In the short term, it enhances postprandial glucose tolerance and β-cell function; in the medium term, it lowers HbA1c, reduces insulin resistance, and improves body composition; and in the long term, it enhances insulin sensitivity, preserves muscle mass, and produces sustained metabolic benefits. Dietary weight loss and energy restriction further amplify these effects, while environmental modifiers such as hypoxia appear to have minimal additional impact.

### 3.2 Resistance exercise

Resistance exercise, which involves the active contraction of muscles against external resistance, requires less cardiorespiratory endurance than aerobic exercise and is an effective approach for improving muscle strength, mass, and endurance. In the elderly population, resistance training is widely applied as a primary intervention to counteract sarcopenia, and several studies have also reported beneficial effects on glucose metabolism and insulin sensitivity. However, the available evidence is not entirely consistent, suggesting that the metabolic outcomes of resistance exercise may depend on multiple factors, including intervention duration, training intensity, baseline metabolic status, and dietary context.

Evidence from medium-term interventions (8–12 weeks) more consistently supports the metabolic benefits of resistance training. For example, 12 weeks of elastic band training achieved high adherence rates (95%) among obese older women and led to significant reductions in blood glucose, insulin, HOMA-IR, body fat, and waist circumference, while simultaneously increasing lean body mass (Son and Park, 2021). Similarly, progressive resistance training improved not only muscle strength but also waist circumference, fasting glucose, basal insulin levels, and insulin resistance in older women (Oliveira et al., 2015), with additional reductions in glucose and waist circumference observed in women with metabolic syndrome (Tomeleri et al., 2018). In older adults with T2D, 12 weeks of resistance training reduced intermuscular fat, increased muscle mass, and improved β-cell function and early-phase insulin secretory response (Tang et al., 2024). Resistance training has also been shown to reverse metabolic impairments caused by physical inactivity. For instance, older adults who experienced marked declines in insulin sensitivity following short-term bed rest regained baseline insulin sensitivity after an eight-week eccentric exercise program, accompanied by improvements in muscle strength and hypertrophy (Reidy et al., 2018). Collectively, these findings indicate that resistance training can improve both glucose metabolism and body composition, particularly in older individuals without severe comorbid metabolic disease.

Findings from long-term interventions (≥6 months) are less consistent. Some studies have reported that 6 months of moderate-intensity resistance training significantly enhanced β-cell secretory function and reduced intermuscular fat in patients with T2D (Tang et al., 2024). However, other trials found no significant improvements: 6 months of strength training failed to enhance insulin sensitivity or muscle glycogen content in older men (Jensen et al., 2018), and 12 months of continuous high-intensity resistance training in patients with T2Ddid not significantly improve glycemia, HbA1c, or HOMA2-IR. These null results may be partly explained by relatively good baseline glycemic control (HbA1c ∼7.1%) and the confounding effects of medication use in these cohorts (Mosalman Haghighi et al., 2021).

The influence of dietary context and population heterogeneity further complicates interpretation. In a five-month randomized trial, the addition of caloric restriction (∼600 kcal/day) to resistance training resulted in significant reductions in body weight and fat mass but did not produce additional improvements in glycemia or insulin sensitivity beyond those achieved by resistance training alone (Normandin et al., 2017). Population-specific responses have also been reported. In older men with and without T2D, resistance training significantly improved muscle strength in both groups, but reductions in HOMA-IR were observed only in the non-diabetic participants. This suggests that individuals with diabetes may require multimodal interventions, combining resistance training with other exercise modalities or therapies, to achieve optimal metabolic outcomes (Shabkhiz et al., 2021).

Overall, resistance training consistently improves muscle strength and body composition in older adults and has demonstrated beneficial effects on glucose regulation and insulin sensitivity in some studies. Nevertheless, results from longer-term interventions are more variable, particularly in patients with diabetes, and appear to be influenced by baseline glycemic control, pharmacological treatment, training intensity, and individual variability. Further research is needed to optimize resistance training prescriptions and to evaluate combined protocols—such as resistance training integrated with dietary strategies or other exercise modalities—to maximize improvements in glucose metabolism among elderly populations.

### 3.3 HIIT

HIIT can be characterized as intermittent exercise bouts performed above the heavy-intensity domain, interspersed with short recovery periods at low intensity or complete rest (Coates et al., 2023). This domain boundaries are demarcated by indicators that primarily include the critical power or critical speed, or other indices, including the second lactate threshold, maximal metabolic steady state, or lactate turnpoint (Coates et al., 2023). By markedly increasing exercise intensity while reducing total duration, HIIT provides a time-efficient alternative to traditional endurance training. The intermittent structure can delay the onset of discomfort and improve tolerability and adherence among older adults. However, achieving true high-intensity workloads may be difficult for elderly individuals, especially those with cardiovascular, orthopedic, or frailty-related limitations. This highlights the importance of individualized exercise prescription, gradual progression, and medical supervision to ensure safety. Despite these considerations, accumulating evidence indicates that appropriately tailored HIIT protocols can effectively improve cardiorespiratory fitness, body composition, and glucose metabolism in elderly populations, although findings across studies are not fully consistent.

Several intervention trials support the efficacy of HIIT in improving glucose regulation and related risk factors within relatively short timeframes. For example, a 12-week program consisting of two HIIT sessions per week significantly reduced blood glucose and waist circumference in older adults, with a 75% reduction in diabetes prevalence among women, highlighting its potential to reverse glucose metabolism abnormalities (De Matos et al., 2021). Importantly, the benefits may extend beyond insulin sensitivity alone. Acute HIIT protocols—such as four 4-minute intervals or ten 1-minute intervals—have been shown to significantly enhance β-cell insulin secretory function in postmenopausal women, suggesting that HIIT directly improves β-cell responsiveness to glucose stimulation and thus provides a critical physiological mechanism for diabetes management (Low et al., 2025).

Comparisons with moderate-intensity continuous training (MICT) further illustrate the potential advantages of HIIT. An 8-week non-weight-bearing HIIT program in sedentary older adults significantly reduced HOMA-IR and improved insulin sensitivity, whereas no comparable changes were observed with MICT. Additionally, improvements in maximal oxygen uptake were accompanied by parallel increases in cardiac ejection fraction, with a strong correlation between the two outcomes (r = 0.57, P < 0.0001) (Hwang et al., 2016). In older patients undergoing post-myocardial infarction rehabilitation, HIIT produced greater reductions in waist circumference, fasting glucose (−25.8 vs. −3.9 mg/dL, P < 0.001), triglycerides (−67.8 vs. −10.4 mg/dL, P < 0.001), and diastolic blood pressure compared to MICT, while simultaneously reducing adiposity and increasing lean mass (Dun et al., 2019).

Nonetheless, not all studies have demonstrated clear superiority of HIIT over MICT. In a 2-week trial, HIIT produced greater improvements in weight loss and aerobic capacity but yielded comparable benefits in postprandial glycemic control and insulin sensitivity relative to MICT (Malin and Syeda, 2024). Similarly, in a 16-week intervention in older adults with metabolic syndrome, HIIT three times per week (17 or 38 min per session) improved maximal oxygen uptake and reduced central obesity more effectively than MICT, but did not significantly affect fasting insulin, HOMA-IR, glycemia, or lipid profiles. The investigators suggested that the absence of dietary and energy intake restrictions may have attenuated the metabolic benefits of training (Von Korn et al., 2021).

HIIT shows strong potential for improving glucose metabolism, β-cell function, and body composition in older adults, particularly in the short term. Compared with MICT, HIIT often demonstrates superior benefits for cardiovascular fitness and certain metabolic outcomes, although results vary across studies. Differences may reflect heterogeneity in intervention duration, dietary control, baseline metabolic status, and sample size. Large-scale, long-term trials with standardized dietary control are needed to better define the role of HIIT in managing glucose metabolism disorders in the elderly.

### 3.4 Combined exercise training

#### 3.4.1 Effects of combined exercise training

Numerous studies have shown that combined exercise training improves glucose metabolism in older adults across different populations and intervention periods. In both healthy and metabolically impaired individuals, 12 weeks of combined exercise training (e.g., elastic band exercises with walking) effectively reduced blood glucose levels and insulin resistance (Ha and Son, 2018). The underlying mechanism may involve exercise-induced reductions in circulating free fatty acids and the activation of glucose metabolism–related enzymes and receptors, thereby facilitating glucose uptake and utilization (Ha and Son, 2018).

In older women with diabetes, 12 weeks of combined exercise training reduced body weight and fat percentage while increasing muscle mass. However, the improvements in HbA1c and HOMA-IR were modest, suggesting that long-term glycemic markers such as glycated hemoglobin may require longer interventions for significant changes (Jeon et al., 2020). Similarly, in obese older men, 12 weeks of combined exercise training (elastic band plus moderate-intensity aerobic exercise) reduced insulin levels and HOMA-IR, while also improving erythrocyte deformability, decreasing aggregation, and enhancing aerobic capacity. These results indicate that combined exercise training may also promote glucose metabolism indirectly by improving microcirculation and peripheral glucose utilization (Kim et al., 2019).

Even in the context of reduced insulin sensitivity caused by bed rest, combined exercise training composed of aerobic, resistance, and high-intensity interval sessions maintained stable Matsuda index values, highlighting its protective effect against bed rest–related metabolic decline (Mastrandrea et al., 2025). Other studies have shown that 4–6 months of combined exercise training improves glycemia, insulin resistance, body fat distribution, maximal oxygen uptake, and cardiovascular function in older women and patients with multiple sclerosis, demonstrating both metabolic and cardiovascular benefits (Čížková et al., 2020; Braggio et al., 2025).

Mechanistic studies suggest that the enhancement of insulin sensitivity by combined exercise training is primarily mediated by reductions in abdominal fat, especially subcutaneous fat, rather than improvements in cardiorespiratory fitness alone. Abdominal fat and body mass index serve as important mediators between exercise and insulin sensitivity (Ko et al., 2016). In addition, whether exercise was performed in a fasted or postprandial state did not alter the benefits: 8 weeks of combined exercise training significantly reduced HbA1c, fasting insulin, and HOMA-IR, while improving cardiorespiratory fitness and body composition in both conditions, underscoring the robustness of this intervention (Brinkmann et al., 2019). The training schedule also influences outcomes. When resistance and HIIT were performed on the same day, the benefits were smaller compared to split-day training, which produced greater improvements in insulin resistance, fasting glucose, HbA1c, lipid profiles, and cardiorespiratory fitness (Ghodrat et al., 2022).

#### 3.4.2 Combined exercise training versus single-modality exercise

Compared with single-modality exercise, combined exercise training often produces broader and more comprehensive improvements in glucose metabolism; however, this superiority is outcome-dependent and influenced by program composition, total training load, and adherence. For example, while HIIT combined with daily walking improved mitochondrial function and glucose utilization beyond HIIT alone (Mensberg et al., 2025), direct comparisons show that all three modalities (aerobic, resistance, combined) can improve insulin sensitivity, with combined training showing particular advantages for insulin secretory function and reductions in visceral and intermuscular fat—effects that have been linked mechanistically to modulation of CNTFRα and IGF-1 (Colleluori et al., 2025).

The relative contribution of aerobic and resistance components also affects efficacy. In older adults with multiple sclerosis, both aerobic-dominant and resistance-dominant combinations improved glucose metabolism, but aerobic-dominant training yielded greater improvements in blood glucose and lipid levels (Zhou et al., 2022). Other studies have confirmed that combined exercise training is more effective than resistance training alone in reducing fasting insulin and HOMA-IR, even without major changes in body composition (Kim et al., 2018). In women with metabolic syndrome, 20 weeks of combined exercise training (balance, strength, and aerobic exercise) and elastic band resistance training both improved glycemia, lipid profiles, body composition, and physical function. However, combined exercise training provided additional benefits in balance and inflammation reduction, whereas resistance training was more effective in improving lower-limb strength and reducing fat percentage (Gargallo et al., 2024).

These observations highlight two important considerations. First, some apparent advantages of combined training may reflect higher total exercise volume or a greater cumulative physiological stimulus when modalities are added rather than a unique synergistic effect *per se*; studies that match total energy expenditure or time between arms are therefore critical to disentangle modality synergy from volume effects. Second, feasibility and adherence represent critical factors: because combined training often demands more time and coordination, it may be less sustainable for older adults, ultimately limiting its practical impact. Thus, exercise prescriptions for older individuals should be goal-oriented and pragmatic—selecting combined training when the objective is broad metabolic restoration (including β-cell secretory function and ectopic fat reduction), but prioritizing modality choice, intensity, or time-matched designs when resources, adherence, or specific outcomes (such as lower-limb strength) dictate. An overview of the effects of different exercise modalities on glucose metabolism, body composition, and oxygen uptake in older adults is summarized in Table 1. Future trials should report time-matched comparisons and adherence metrics to clarify whether combined training confers intrinsic superiority beyond volume and to determine optimal, implementable regimens for older populations.

**TABLE 1:** Effects of various exercise modalities on glucose metabolism, body composition, and oxygen uptake in older adults.

Participant	Age	Exercise type	Exercise program	Intervention period	Effects on glucose metabolism	Effects on body composition and oxygen uptake	References
Middle-aged and Older Adults	45–80 years	AE; walking/jogging	50%–60% HRR (30–40 min) → 70%–80% HRR (50 min)	6 months, 3×/week	—	VO_2_max ↑; muscle mass ↑ intramuscular fat ↓	Ryan et al. (2021)
Older Adults	60–80 years	AE; walking, cycling, elliptical, rowing	45 min/session (180 min/week)	6 months, 4–5×/week	Insulin sensitivity ↑ Cardiometabolic risk ↓	Weight ↓ BMI ↓	Brennan et al. (2020)
Older adults	62 ± 2 years	AE; treadmill, cycle, elliptical	65% → 85% HR, 45 min/session	12 weeks, 3×/week	HbA1c ↓ Fasting insulin ↓ HOMA-IR ↓ Matsuda index ↑	VO_2_max ↑	Konopka et al. (2019)
Elderly obese	60.9 ± 1.4 years	AE; cycling	60 min, 70% HRpeak	13 days, 12 sessions	120-min and 180-min tAUC of postprandial glucose and insulin ↓; Systemic and adipose tissue IR ↓	BMI ↓ Fat-free mass ↓VO_2_peak (L/min) ↑	Gilbertson et al. (2018)
Postmenopausal Breast Cancer Survivors	35–70 years	AE; Treadmill/bike/elliptical	45–60 min, 65%–85% HRmax	12 weeks, 3×/week	OGTT 120-min insulin ↓	Body weight ↓ CRF ↑	Viskochil et al. (2020)
Elderly obese	66.1 ± 4.4 years	AE; Treadmill or cycle ergometer	60%–65% → 80%–85% HRmax, 50–60 min	12 weeks, 5×/week	FBG ↓ Clamp-measured peripheral IS ↑	Body weight ↓ BMI ↓ VO_2_max ↑	Erickson et al. (2019)
T2D patients	65.3 ± 1.7 years	AE; Interval walking	3-min slow (54% VO_2_max) and 3-min fast (89% VO_2_max) cycle	2 weeks, 10 times	β-cell glucose sensitivity ↑	—	Karstoft et al. (2017)
Postmenopausal women	60.87 ± 5.73	AE; fitness walk	60 min, 50%–60% VO_2_max	12 weeks, 5×/week	TG ↓; HDL-C ↑; Blood pressure ↓	Waist circumference ↓	Li et al. (2025)
Sedentary elderly	62 ± 6 years	AE; cycling	W1-4: 60% VO_2_peak for 30 min; W5-8: 70% VO_2_peak for 40 min	8 weeks, 3×/week	IS (GIR/M, HOMA-IR, ISI) ↑	Sub-minimal fat oxidation ↑	Chobanyan-Jürgens et al. (2019)
Postmenopausal women, T2D	60–69 years	AE; walking or running	45 min, 60%–70% HRR	12 weeks, 3×/week	HbA1c ↓; FBG ↓; HOMA-IR ↓	Body weight ↓; BMI ↓	Tan et al. (2018)
Elderly patients with T2D and comorbidities	71.6 ± 5.6 years	RT; machines	45 min/session, 80% 1RM or RPE 15–18	12 months, 3×/week	HbA1c ↑; IS ↑; 48-h continuous glucose ↓	—	Simpson et al. (2015)
Elderly male with/without T2D	65–75 years	RT; Machines/Free Weights	45–60 min/session	12 weeks, 3×/week	Non-T2D: HOMA-IR ↓	Muscle strength ↑	Shabkhiz et al. (2021)
Elderly after bed rest	60–75 years	RT (Rehabilitative); Machines/Free Weights	High-Intensity	8 weeks, 3×/week	Insulin sensitivity ↓	Lower limb muscle hypertrophy restored	Reidy et al. (2018)
Postmenopausal obese women	55–60 years	RT; Resistance Bands	60 min/session, 40%–70% 1RM	12 weeks, 3×/week	Glucose, insulin, HOMA-IR ↓; TG and LDL-C ↓; HDL-C ↑	Body weight, BMI, body fat %, waist circumference ↓; Lean body mass ↑	Son and Park (2021)
Elderly patients with T2D	60–75 years	RT	40 min, moderate Intensity	6 months, 3×/week	β-cell function ↑	intermuscular AT ↓; Normal muscle area ↑	Tang et al. (2024)
Postmenopausal women	65.0 ± 4.2 years	RT	RPE-based intensity	12 weeks, 3×/week	Blood glucose, basal insulin, HOMA-IR ↓; Total cholesterol, LDL-C ↓	Waist circumference ↓; Muscle strength ↑	Oliveira et al. (2015)
Overweight/obese elderly	65–79 years	RT	70% 1RM	5 months, 3×/week	FBG ↓	Weight ↓; Fat mass ↓	Normandin et al. (2017)
Older women with MetS	60–79 years	RT	Moderate Intensity, Progressive Overload	6 months, 3×/week	β-cell function (HOMA2-β) ↑; early-phase insulin secretion ↑; 2-h postprandial glucose ↓	intermuscular AT ↓; normal muscle area ↑	Gargallo et al. (2024)
Elderly male ± T2D	63–73 years	RT	Gradual increase in resistance	12 weeks, 3×/week	HOMA-IR ↓; FGF-21 ↓; Myostatin ↓	Muscle strength ↑	Jensen et al. (2018)
Older women	70.4 ± 5.7 years	RT (Rehabilitative); Machines/Free Weights	High-Intensity	8 weeks, 3×/week	IS ↑	Muscle macrophage content ↑	Tomeleri et al. (2018)
Older adults	60–79 years	RT (Rehabilitative); Machines/Free Weights	90 min/session	12 weeks, 2×/week	Blood glucose ↓; insulin ↓; HOMA-IR ↓	Body weight ↓; BMI ↓; body fat % ↓; lean body mass ↑	Agner et al. (2018)
Sedentary Healthy Older Adults	65 ± 1 years	HIIT; Whole-body ergometer	28 min/session, 90% HRpeak	8 weeks, 4×/week	HOMA-IR ↓	—	Hwang et al. (2016)
Pre-diabetic elderly	60.8 ± 11.3 years	HIIT	3 sets of 20-s sprints with recovery	12 weeks, 3×/week	Peripheral and whole-body IS ↑	—	Mensberg et al. (2025)
Patients with MetS	61 ± 5 years	HIIT	17 min/session, 80%–90% HRR	16 weeks, 3×/week	Fasting glucose, insulin, HOMA-IR, blood lipids →	VO_2_peak and RER ↑	Von Korn et al. (2021)
Pre-diabetic elderly	60.3–60.8 years	HIIT; Cycle Ergometer	3-min high/3-min low intensity, 90% HRpeak	12 sessions over 13 days	IS ↑; postprandial glucose ↓	BMI ↓; VO_2_peak ↑	Malin and Syeda (2024)
Postmenopausal women with T2D	70 ± 5 years	HIIT	25 min (4x4-min HI + 3x3-min recovery)	Single Session	β-cell glucose sensitivity ↑	—	Low et al. (2025)
Patients with MetS post-MI	68 ± 10 years	HIIT	4–8 intervals of 30–60s, RPE 15–17	Completion of 36 sessions	MetS risk ↓; FBS ↓; TG ↓	Waist circumference ↓; Fat mass ↓; Lean mass ↑	Dun et al. (2019)
Older adults	—	HIIT; Treadmill Running	40 min/session (inc. cool-down), 85%–90% HRmax	12 weeks, 2×/week	glucose levels ↓	MetS parameters ↓	De Matos et al. (2021)
Older adults	70–80 years	CT (AE + RT); Bands + Walking	50 min/session (20-min RT + 30-min walk) progressed to 60%–70% HRR	12 weeks, 3×/week	Blood glucose ↓	—	Ha and Son (2018)
Postmenopausal women with T2D	62.1 ± 7.3 years	CT (AE + RT); Dance + Bands	50 min/session (20-min AE + 30-min RT)	12 weeks, 3×/week	HOMA-IR ↓; HDL-C ↑	Body weight ↓; body fat % ↓; total fat mass ↓; ASM/weight ratio ↑	Jeon et al. (2020)
Obese older men	68.8 ± 0.9 years	CT (AE + RT); Bands + Treadmill/Bike	90–100 min/session (30–40-min RT + 60-min AE)	12 weeks, 3×/week	HOMA-IR ↓	Body fat percentage ↓	Kim et al. (2019)
Healthy elderly adults	55–65 years	CT (AE + RT + HIIT)	60 min/day, HIIT every other day	14 days	HOMA-IR ↑; Glucose/insulin AUC ↑	body weight ↓	Mastrandrea et al. (2025)
Healthy/active older women	62–80 years	CT (AE + RT)	Progressive Intensity	4 months, 3×/week	Glucose ↓; HbA1c ↓	Body fat % ↓; waist circumference ↓; Muscle fat metabolism ↑	Čížková et al. (2020)
Multiple sclerosis patients	63 ± 9.4 years	CT (AE + RT)	Moderate Intensity	6 months	Fasting glucose ↓	Body weight ↓; waist circumference slightly ↓	Braggio et al. (2025)
Sedentary elderly (MetS)	60–80 years	CT (AE + RT)	60%–75% VO_2_peak	6 months, 3×/week	IS ↑; HOMA-IR ↓	Visceral AT ↓; subcutaneous AT ↓; Waist circumference ↓; BMI ↓	Ko et al. (2016)
T2D	60 ± 8 years	CT (AE + RT)	30 min/session	8 weeks, 3×/week	HbA1c ↓; serum insulin ↓; HOMA-IR ↓	Body fat ↓; fat-free mass ↑	Brinkmann et al. (2019)
T2D	45–65 years	CT (HIIT + RT)	40%–70% 1RM; 60%–90% VO_2_max	8 weeks	HOMA-IR ↓; fasting glucose ↓; fasting insulin ↓; HbA1c ↓	Fat mass ↓; fat-free mass ↑; VO_2_max ↑	Ghodrat et al. (2022)
Obese elderly	≥65 years	CT (AE + RT)	75–90 min of cardio and resistance training	6 months, 3×/week	HOMA-IR ↓	Disposition index (DI) ↑; body weight ↓	Colleluori et al. (2025)
Elderly patients with MetS	≥60 years	CT (AE-focused); CT (RT-focused)	50 min/session	12 weeks, 3×/week	FPG ↓; 2hPG ↓; Blood lipids ↓	BMI ↓	Zhou et al. (2022)
Postmenopausal Diabetic Women	62.1 ± 7.3 years	CT (AE + RT)	50 min/session	12 weeks, 3×/week	Fasting insulin ↓; HOMA-IR ↓	Upper Body Muscle Strength ↑	Kim et al. (2018)

AE, aerobic exercise; RT, resistance training; HIIT, High-Intensity Interval Training; CT, combined training; T2D = Type 2 Diabetes; MetS = metabolic syndrome; HRR, heart rate reserve; HRmax, Heart Rate Maximum; 1RM, One-Repetition Maximum; RPE, rate of perceived exertion; VO_2_max/peak = Maximal/Peak Oxygen Uptake; HOMA-IR, homeostatic model assessment of insulin resistance; IS, insulin sensitivity; FBG, fasting blood glucose; HbA1c = Glycated Hemoglobin; OGTT, oral glucose tolerance test; AUC, area under the curve; tAUC, total AUC; TG, triglycerides; HDL-C, High-Density Lipoprotein Cholesterol; LDL-C, Low-Density Lipoprotein Cholesterol; AT, adipose tissue; BMI, body mass index; CRF, cardiorespiratory fitness; RER, respiratory exchange ratio; ASM, appendicular skeletal muscle; FGF, fibroblast growth factor; DI, disposition index; FPG, fasting plasma glucose; 2hPG, 2-h Postprandial Glucose; IR, insulin resistance; ↑ = Increase; ↓ = Decrease; → = Unchanged;/= not reported.

## 4 Exercise dose and prescription for different intervention types in glucose metabolism in older adults

### 4.1 Aerobic exercise

Several studies have quantified aerobic exercise intensity using either percentage of heart rate reserve (HRR) or maximum heart rate (HRmax). For older adults, intensity should be prescribed progressively, with low to moderate levels as the most tolerable and safe starting point.

Walking or jogging for 30–40 min, three times per week at 50%–60% HRR—gradually increasing to 65%–75% HRR over 4 weeks—has been shown to significantly reduce the late-phase insulin secretion curve during OGTT (120–180 min), indicating improvements in both β-cell function and tissue insulin sensitivity (Ryan et al., 2021). Similarly, fitness walking at 50%–60% VO_2_max (60 min, five times per week) did not significantly alter fasting glucose or Body Mass Index (BMI) (Li et al., 2025), but it provided an adaptive foundation for progressing to higher-intensity exercise. Notably, insufficient intensity can limit efficacy, as observed in the low-intensity walking program described above (Li et al., 2025).

Moderate-to-high intensity exercise appears to be the key driver of substantial metabolic benefits. For example, a tapering protocol beginning at 60%–65% HRmax and progressing to 80%–85% HRmax over 4 weeks (50–60 min, five times per week) significantly improved peripheral insulin sensitivity and VO_2_max (Erickson et al., 2019). Another study using 45-minute sessions three times per week—15 min at 60% HRmax followed by 30 min at target intensity (progressively increased from 65% to 85% HRmax)—led to significant reductions in HbA1c, fasting insulin, whole-body adiposity, and postprandial glucose fluctuations after 12 weeks (Konopka et al., 2019).

Individualized prescriptions can further optimize outcomes. For instance, a protocol based on FATmax, determined via gas exchange analysis, produced significant reductions in visceral adiposity and insulin resistance in older women with T2Dafter 12 weeks. The intervention consisted of 60 min of training three times per week, including 20–40 min at target intensity (Tan et al., 2018). This suggests that tailoring exercise dose to physiological metrics may be more effective than applying fixed percentage-based prescriptions. Beyond protocols based on FATmax, recent methodological advances emphasize the limitations of percentage-based intensity prescriptions. Studies have shown that the large ranges in both sexes at which lactate threshold and maximal lactate steady-state occurred on the basis of %VO_2_max, %WRpeak, and %HRmax elicited large variability in the number of individuals distributed in each domain at the fixed-percentages examined (Iannetta et al., 2020). Consequently, there is a shift toward domain-based prescription, in which exercise intensity is defined by directly measured physiological boundaries such as lactate thresholds (Inglis et al., 2024). Application of this approach would optimize health-related outcomes of participants and better characterize the molecular and system-level adaptations related to acute and chronic exercise trainings (Iannetta et al., 2020). For older adults, this strategy can improve both precision and safety in exercise dosing.

In most studies, aerobic exercise is prescribed at a frequency of 3–5 sessions per week. Three 45-min sessions per week, with intensity tapering from 65% to 85% HRmax, significantly improved HbA1c, fasting insulin, and adiposity in pre-diabetic older adults (Konopka et al., 2019). By contrast, low-intensity walking (50%–60% VO_2_max, 60 min, five times per week) did not significantly change fasting glucose or BMI, indicating that frequency and duration cannot compensate for insufficient intensity (Li et al., 2025).

Finally, for older adults, exercise prescription should also consider variety and feasibility. Treadmill walking/jogging, stationary cycling, and elliptical training are commonly used modalities (Ryan et al., 2021; Konopka et al., 2019; Viskochil et al., 2020). Allowing participants to alternate between these options based on preference has been shown to improve adherence (Konopka et al., 2019).

### 4.2 Resistance exercise

Resistance training has been widely demonstrated to improve glucose metabolism abnormalities in older adults, but its effects vary depending on training intensity. Using the percentage of one-repetition maximum (1RM) as a criterion, interventions can be categorized into low-intensity (<50% 1RM), moderate-intensity (50%–70% 1RM), and high-intensity (≥70% 1RM), which provides clearer insight into the dose–response relationship between resistance training and improvements in glucose metabolism.

From the perspective of feasibility and adherence, resistance training with elastic bands represents a safe, executable, and effective entry-level option for older adults, particularly those with obesity or metabolic syndrome. For example, older women training at 40%–50% 1RM for approximately 60 min, three times per week, achieved significant reductions in blood glucose, insulin resistance, and body fat percentage, alongside increases in lean body mass after 12 weeks (Son and Park, 2021). Similarly, a 6-month program starting at 45% 1RM and progressively increasing in intensity reduced intermuscular fat deposition and improved β-cell function (Tang et al., 2024). These findings suggest that although low-intensity training produces slower-onset benefits, when performed over sufficient duration it can positively influence glucose metabolism and body composition, making it particularly suitable for individuals with low baseline fitness or limited initial exercise compliance. Moreover, for older adults unable to tolerate moderate-to high-intensity resistance training, blood flow restriction training (BFRT) represents a promising alternative. Meta-analytic evidence in overweight/obese adults indicates that BFRT combined with low-load RT significantly improves FBG and HOMA-IR compared with RT alone (Chen et al., 2025). Thus, BFRT may serve as a safe, feasible entry-level resistance training strategy for frail older adults or those with comorbidities, offering meaningful metabolic benefits when conventional moderate-intensity RT is not feasible.

Evidence for moderate-intensity resistance training is the most consistent. Typical protocols involve three sessions per week, covering 8–10 full-body exercises, with 8–12 repetitions per set, performed at 55%–70% 1RM for 12–24 weeks (Oliveira et al., 2015; Tomeleri et al., 2018; Normandin et al., 2017; Shabkhiz et al., 2021). These interventions generally report reductions in blood glucose, fasting insulin, and HOMA-IR, alongside decreases in waist circumference and body fat, as well as gains in muscle strength. Importantly, while non-diabetic older adults can show improvements in insulin resistance after as little as 12 weeks, patients with T2Doften require longer training durations (≥6 months) or higher loads to achieve comparable metabolic benefits (Shabkhiz et al., 2021). Thus, moderate-intensity training provides an optimal balance of safety, adherence, and efficacy, explaining why it is often recommended as the preferred intensity range for older adults with impaired glucose metabolism.

Findings for high-intensity resistance training are more heterogeneous. On one hand, eccentric resistance training combined with protein supplementation fully restored insulin sensitivity in older adults after just 8 weeks of rehabilitation following 5 days of bed rest, and was accompanied by muscle hypertrophy and strength gains (Reidy et al., 2018). On the other hand, long-term high-intensity protocols (70%–80% 1RM for 6–12 months) failed to significantly improve blood glucose, HbA1c, or HOMA-IR in some trials (Jensen et al., 2018; Mosalman Haghighi et al., 2021). Potential explanations include participants’ already well-controlled baseline glucose metabolism, which left little room for improvement, or confounding factors such as concurrent medication use masking the effects of training. These findings suggest that while high-intensity training is uniquely valuable for restoring function and rapidly enhancing muscle strength, its glycometabolic benefits are less consistent.

### 4.3 HIIT

In older adults, HIIT is emerging as a particularly effective modality for improving glucose metabolism, showing a clear gradient effect of “intensity dosage.” Programs that reach near-maximal effort (≥85% HRmax or HRpeak) have consistently demonstrated substantial benefits. For example, a 12-week, twice-weekly treadmill HIIT protocol (40 min per session, consisting of 10 sets of 1-minute sprints at 85%–90% HRmax with 1-minute walking recovery intervals) significantly reduced blood glucose levels and waist circumference, and decreased the prevalence of diabetes by approximately 75% in older men and women (De Matos et al., 2021). Similarly, a non-weight-bearing, whole-limb cycling HIIT program (four sessions per week, each consisting of 4 × 4 min at 90% HRpeak with 3-minute intervals at 70% HRpeak; ∼25 min total) produced significant reductions in HOMA-IR, improved insulin sensitivity, and increased VO_2_max, which was strongly correlated with improvements in cardiac ejection fraction (Hwang et al., 2016). These findings suggest that high-intensity intervals (≥85% HRmax/HRpeak) are effective for enhancing both insulin sensitivity and cardiorespiratory fitness, and are closely linked to improved glucose metabolism. Importantly, even acute sessions of HIIT have shown effects: single bouts of 4 × 4-minute or 10 × 1-minute protocols significantly improved β-cell glucose sensitivity in postmenopausal women with T2D, with increases of 15–16 mmol/L (Low et al., 2025).

By contrast, when HIIT is performed at relatively lower intensities or with insufficient total training load, its metabolic benefits are less consistent. For instance, a 2-week high-frequency protocol (12 sessions over 13 days, alternating intervals at 90% HRpeak and 50% HRpeak, ∼60 min per session) significantly improved body weight and aerobic capacity but failed to produce superior effects on fasting glucose or insulin sensitivity compared with higher-intensity regimens (Malin and Syeda, 2024). Similarly, a 16-week intervention with two HIIT formats (17 min or 38 min per session, three times per week, alternating 4-minute intervals at 80%–90% HRR with 3-minute recovery at 35%–50% HRR) improved VO_2_max and central adiposity but did not significantly alter fasting insulin, HOMA-IR, or glycemia (Von Korn et al., 2021). These outcomes suggest that metabolic improvements may require not only sufficient intensity but also adequate overall training volume and, in some cases, dietary control to reinforce exercise-induced adaptations.

Notably, when prescribing exercise for older adults, baseline frailty, mobility limitations, and common co-morbidities such as obesity, osteoarthritis, or metabolic syndrome can substantially influence both the choice and effectiveness of exercise modalities. Low-intensity aerobic exercise, such as walking at 50%–60% HRR for 30–60 min, 3–5 times per week, can improve β-cell function and insulin sensitivity even in participants with limited fitness or mobility (Ryan et al., 2021), while providing a safe foundation for progressing to moderate-to-high intensity protocols (Li et al., 2025). Similarly, resistance training using elastic bands at low-to-moderate intensity (40%–50% 1RM, 3 sessions/week) offers a feasible entry point for frail older adults or those with joint limitations (Son and Park, 2021). HIIT confers substantial improvements in insulin sensitivity and cardiorespiratory fitness when ≥85% HRmax/HRpeak is achieved (De Matos et al., 2021; Hwang et al., 2016), but for frail or orthopedic/cardiac-impaired individuals, lower-intensity or shorter-interval protocols may be safer. Overall, exercise prescriptions should be carefully tailored to individual functional capacity, co-morbidities, and tolerance, with progression guided by adaptation, safety, and adherence.

### 4.4 Exercise combined with pharmacotherapy

Exercise and pharmacotherapy are two cornerstone interventions for managing diabetes in older adults and are frequently used in combination (Xiao et al., 2025). However, the interactions between medications and exercise are complex, and their effects are not simply additive.

Research indicates that both exercise and metformin enhance insulin sensitivity by activating the AMPK signaling pathway in skeletal muscle, making them effective strategies for preventing the progression from prediabetes to diabetes. However, their combined use can yield complex outcomes. A 12-week intervention study found that although both exercise training and metformin significantly improved insulin sensitivity in individuals with prediabetes, data from the combined group suggested that metformin may attenuate the full beneficial effects of exercise training alone (Malin et al., 2012). Subsequent research further confirmed that metformin antagonizes exercise-induced improvements in insulin sensitivity and cardiorespiratory fitness (e.g., VO_2_max) (Konopka et al., 2019). At the molecular level, metformin attenuates resistance training-induced activation of the mTORC1 signaling pathway, potentially impairing the muscle hypertrophy response in older adults (Walton et al., 2019). Concurrently, it abolishes exercise-mediated enhancements in skeletal muscle mitochondrial respiratory function (Konopka et al., 2019). Therefore, although metformin is a first-line diabetes treatment and a potential anti-aging agent, these adverse effects on exercise adaptations necessitate careful evaluation of its impact before widespread use in older adults.

In contrast to metformin, SGLT-2 inhibitors exhibit synergistic potential when combined with exercise. Both exercise and SGLT2i independently improve glycemic parameters, and their combination leads to further significant improvements in glucose tolerance and insulin response (Linden et al., 2019). Regarding exercise capacity, the combined therapy not only caused no deterioration but also resulted in superior submaximal exercise capacity in animal models, evidenced by a significantly increased running distance to fatigue, alongside notable weight reduction (Linden et al., 2019). The underlying mechanism may involve SGLT2i promoting greater reliance on fat as an energy source during exercise. Furthermore, as an adjunct to diet and exercise, the bile acid sequestrant colesevelam exhibits both glucose- and lipid-lowering effects, with a good safety profile in older patients that does not increase hypoglycemia risk and may reduce the burden of polypharmacy (Marrs, 2012).

In summary, different drug classes produce distinct effects when combined with exercise. When developing treatment plans for older adults with diabetes, it is essential to consider their hepatic and renal function, comorbidities, and potential drug interactions to ensure an individualized approach.

## 5 Possible mechanisms of exercise to improve glucose metabolism disorders in the elderly

**FIGURE 1:**
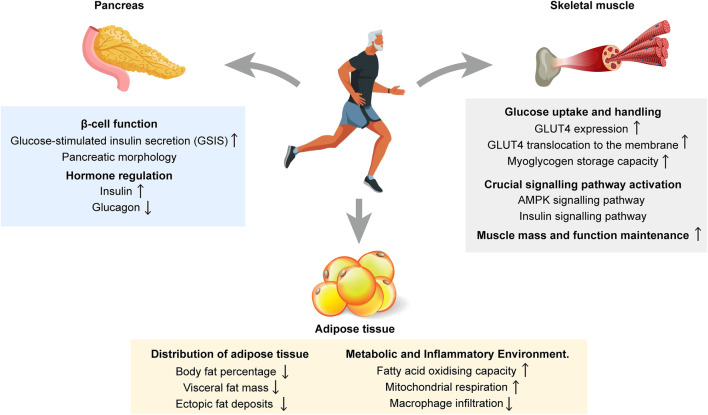
Schematic illustration of the multi-organ mechanism by which exercise ameliorates aging-associated disorders of glucose metabolism. Exercise effectively improves glucose metabolism disorders by acting on three key organs and tissues, namely skeletal muscle, adipose tissue and pancreatic β-cells, and integrating multiple physiological and biochemical pathways, which ultimately work together to enhance insulin sensitivity and maintain glucose homeostasis.

As a non-pharmacological strategy to improve glucose metabolism disorders in the elderly, the mechanism of exercise intervention involves the synergistic regulation of multiple organs and systems. Specifically, exercise enhances insulin sensitivity and glucose homeostasis by targeting key organs, such as skeletal muscle, adipose tissue, and pancreatic β-cells, and by integrating multiple pathways, including energy metabolism, endocrine regulation, and inflammatory response (Figure 1). The specific mechanisms by which exercise affects each tissue are outlined below.

### 5.1 Skeletal muscle

Deterioration of skeletal muscle mass, metabolic fitness, and contractile vigor is fundamental to the progression of metabolic disease and age-associated loss of independence. Skeletal muscle insulin resistance is a common feature of aging and a strong predictor of metabolic disease progression and muscle strength and mass (St-Jean-Pelletier et al., 2017; Distefano et al., 2017; Okamura et al., 2019). Muscle is the primary target tissue for insulin-stimulated glucose disposal and a key regulator of whole-body glucose homeostasis. Thus, reduced muscle mass is also associated with reduced energy expenditure (Fealy et al., 2021). A central mechanism by which exercise improves glucose metabolism disorders is through its direct modulation of glucose transport and processing in skeletal muscle. Studies have shown that exercise training improves glucose regulation in older men by enhancing both the capacity and acute regulation of glucose uptake, as well as by promoting intracellular glucose removal for glycogen synthesis rather than glucose oxidation (Biensø et al., 2015). Furthermore, exercise, particularly aerobic and resistance training, increases the expression of GLUT4 and promotes its translocation to the cell membrane, thereby enhancing both insulin-dependent and non-insulin-dependent glucose uptake in muscle. Notably, lifelong physical activity may prevent age-related insulin resistance in human skeletal muscle by increasing glucose transporter protein expression (Bunprajun et al., 2013). Although exercise can increase GLUT4 expression, this is insufficient to improve insulin-stimulated glucose transport in aged rats (Youngren and Barnard, 1985). This suggests the importance of enhancing insulin signaling integrity in the context of aging.

Aged skeletal muscle exhibits impaired mitochondrial energy production (Braggio et al., 2025) and increased mitochondrial-mediated apoptosis (Gouspillou et al., 2014; Chabi et al., 2008). Exercise-induced activation of AMPK signaling plays a critical role, not only as a catalyst for mitochondrial biogenesis, glucose processing, and fatty acid catabolism, but also by temporally coordinating mTOR activity to support muscle maintenance without exacerbating insulin resistance (Mingzheng and You, 2025). In aged, sedentary rats, exercise training significantly improved impairments in anabolic pathways, including insulin signaling, in a dose-dependent manner (Pasini et al., 2012). Additionally, endurance exercise enhances muscle glycogen storage capacity and non-oxidative glucose processing, and strengthens glucose transport and intracellular fixation by increasing the expression of GLUT4 and hexokinase (Wasserman, 2022).

For older adults, exercise training holds particular significance in increasing muscle mass, as it directly targets sarcopenia—a core feature of aging. Maintaining muscle mass is not only crucial for preserving glucose disposal capacity but also serves as a fundamental strategy to counteract age-related declines in metabolic rate and physical function. As a key metabolic organ accounting for 40%–50% of body weight, skeletal muscle plays a major role in glucose processing. Its contraction stimulates glucose uptake independent of insulin and continues to enhance insulin sensitivity post-exercise (Thyfault, 2008). Specifically, resistance training has proven effective in combating sarcopenia and improving muscle mass and metabolic health (Fragala et al., 2019). A meta-analysis showed that resistance training generally enhances strength, body composition, and insulin sensitivity in older adults, despite variations across studies (Chen et al., 2021). Aerobic exercise has also shown benefits: for instance, 6 months of walking/running increased thigh cross-sectional area by 9% in older men (Schwartz et al., 1991). Additionally, regular endurance exercise enhances muscle oxidative capacity and lipid metabolic efficiency by promoting mitochondrial biogenesis, remodeling, and autophagy, optimizing the coordinated utilization of glucose and lipids (Kim et al., 2017). Overall, exercise mitigates disturbances in glucose metabolism and slows the age-related decline in metabolic function through multiple mechanisms, both before and after the onset of sarcopenia.

### 5.2 Adipose tissue

Age-related changes in adipose tissue involve redistribution of deposits and composition, in parallel with the functional decline of adipocyte progenitors and accumulation of senescent cells (Ou et al., 2022). The mechanism by which exercise reduces visceral fat is particularly important for older adults, since ageing is an independent risk factor for its accumulation. Visceral fat is closely associated with insulin resistance in the elderly population (Tchernof and Després, 2013). Studies have shown that approximately 70 min of moderate-intensity exercise per day is effective in preventing long-term weight gain and progression to obesity in older men. Randomized trials have also shown that visceral fat is negatively correlated with aerobic capacity and that exercise-related reductions in total body fat and abdominal fat are strongly associated with a reduced risk of T2Dand cardiovascular disease (Shiroma et al., 2012; Nicklas et al., 2009).

In addition to overall fat loss, exercise promotes intrinsic metabolic changes in adipose tissue. While fat mobilization may be impaired in older adults due to decreased sympathetic responses and β-adrenergic sensitivity, endurance training enhances fatty acid oxidation, likely due to metabolic adjustments in skeletal muscle rather than direct alterations in lipolysis rates. Exercise also significantly reduces ectopic fat deposition, with both high-intensity and resistance training reducing epicardial fat mass and improving pericardial fat accumulation in patients with abdominal obesity (Christensen et al., 2019). Furthermore, long-term endurance exercise increases mitochondrial respiration and reduces macrophage infiltration in adipose tissue (Sahl et al., 2024), while 12 months of combined training induces adipose tissue remodeling (Zaidi et al., 2019). These changes collectively reduce low-grade inflammation, decrease adipogenesis, and improve insulin sensitivity, alleviating glucose metabolism disorders, although the precise mechanisms are not fully understood.

### 5.3 Pancreatic β-cells

Aging progressively impairs insulin secretion by inducing β-cell senescence, downregulating functional genes, losing proliferative capacity, and activating the senescence-associated secretory pattern. However, the specific alterations in insulin secretion are regulated by complex mechanisms and may undergo a compensatory enhancement phase (Lee and Lee, 2022). Exercise significantly improves pancreatic β-cell function, particularly in elderly individuals with impaired glucose tolerance (IGT). Although normal aging is often accompanied by insulin resistance and decreased insulin secretion, short-term exercise has been found to not only improve insulin sensitivity but also enhance β-cell function in elderly IGT patients (Bloem and Chang, 2008). It is important to note that older adults often compensate for insulin resistance in skeletal muscle, liver, and adipose tissue by secreting more insulin and are more prone to lipid metabolism abnormalities than younger individuals.

In terms of specific mechanisms, exercise can regulate insulin secretion in different tissues, promoting glucose-stimulated insulin secretion (GSIS) and favoring the reduction of circulating glucose. However, older adults tend to exhibit adipose insulin resistance and a lack of compensatory elevation of GSIS after exercise, suggesting they are more prone to concurrent dyslipidemia (Malin et al., 2023). Animal studies have demonstrated that exercise improves serum insulin levels and pancreatic morphology in aging rats (Reaven and Reaven, 1981), and a 12-month exercise intervention prevents age-related islet pathology. Clinical studies have also shown that, in obese older adults, exercise therapy improves β-cell function, reduces plasma glucagon levels, and enhances insulin action, thereby effectively reducing the risk of T2D (Villareal et al., 2008). In summary, exercise, as a non-pharmacological intervention, can effectively counteract age-related β-cell dysfunction through multiple mechanisms and improve systemic glucose metabolism. Therefore, maintaining regular physical activity is crucial for preventing and managing T2D in older adults.

## 6 Conclusion

Aging-related glucose metabolism disorder is a progressive, multi-organ, and multifactorial condition caused by reduced skeletal muscle insulin sensitivity, adipose tissue redistribution and inflammation, and gradual decline in β-cell secretory capacity. Evidence shows that aerobic training, resistance training, HIIT, and combined training can all ameliorate these disorders, but with distinct dose–response characteristics and target outcomes. Moderate to high intensity appears essential for aerobic training to achieve meaningful metabolic benefits, while moderate-intensity resistance training (50%–70% 1RM) is generally preferred for older adults with glucose abnormalities, as it balances safety with efficacy. HIIT has strong potential to enhance β-cell function and reduce blood glucose, particularly in the short term, whereas combined training demonstrates the most consistent long-term improvements across multiple metabolic markers and is recommended as the optimal strategy for this population. Mechanistic studies in both clinical and experimental settings indicate that exercise improves glucose metabolism by upregulating GLUT4 expression and translocation in skeletal muscle, enhancing mitochondrial function and autophagy, reducing visceral and ectopic adiposity, suppressing chronic low-grade inflammation, and directly augmenting β-cell glucose-stimulated insulin secretion.

Future studies are warranted to refine the dose–response relationships of different modalities, optimize exercise prescriptions, and elucidate the underlying pathways to maximize the therapeutic benefits of exercise for aging-associated glucose metabolism disorders. Specifically, future trials should directly compare isocaloric doses of aerobic, resistance, interval, and combined training to delineate their relative metabolic efficiency in older adults. Mechanistic investigations employing stable isotope tracer techniques are also needed to precisely quantify hepatic versus peripheral insulin sensitivity in response to different training paradigms, thereby advancing the mechanistic understanding of exercise-induced glucose regulation in aging.

## References

[B1] Agner V. F. C. Garcia M. C. Taffarel A. A. Mourão C. B. da Silva I. P. da Silva S. P. (2018). Effects of concurrent training on muscle strength in older adults with metabolic syndrome: a randomized controlled clinical trial. Arch. Gerontol. Geriatr. 75, 158–164. 10.1016/j.archger.2017.12.011 29306767

[B2] Aguayo-Mazzucato C. (2020). Functional changes in beta cells during ageing and senescence. Diabetologia 63 (10), 2022–2029. 10.1007/s00125-020-05185-6 32894312 PMC7990033

[B3] American Diabetes Association Professional Practice Committee (2025). 2. Diagnosis and classification of diabetes: standards of care in Diabetes-2025. Diabetes Care 48 (1), S27–s49. 10.2337/dc25-S002 39651986 PMC11635041

[B4] Amorim J. A. Coppotelli G. Rolo A. P. Palmeira C. M. Ross J. M. Sinclair D. A. (2022). Mitochondrial and metabolic dysfunction in ageing and age-related diseases. Nat. Rev. Endocrinol. 18 (4), 243–258. 10.1038/s41574-021-00626-7 35145250 PMC9059418

[B5] Ballak D. B. Stienstra R. Tack C. J. Dinarello C. A. van Diepen J. A. (2015). IL-1 family members in the pathogenesis and treatment of metabolic disease: focus on adipose tissue inflammation and insulin resistance. Cytokine 75 (2), 280–290. 10.1016/j.cyto.2015.05.005 26194067 PMC4553099

[B6] Bapat S. P. Myoung S. J. Fang S. Liu S. Zhang Y. Cheng A. (2015). Depletion of fat-resident treg cells prevents age-associated insulin resistance. Nature 528 (7580), 137–141. 10.1038/nature16151 26580014 PMC4670283

[B7] Bellary S. Kyrou I. Brown J. E. Bailey C. J. (2021). Type 2 diabetes mellitus in older adults: clinical considerations and management. Nat. Rev. Endocrinol. 17 (9), 534–548. 10.1038/s41574-021-00512-2 34172940

[B8] Biensø R. S. Olesen J. Gliemann L. Schmidt J. F. Matzen M. S. Wojtaszewski J. F. P. (2015). Effects of exercise training on regulation of skeletal muscle glucose metabolism in elderly men. J. Gerontol. A Biol. Sci. Med. Sci. 70 (7), 866–872. 10.1093/gerona/glv012 25991826

[B9] Bloem C. J. Chang A. M. (2008). Short-term exercise improves beta-cell function and insulin resistance in older people with impaired glucose tolerance. J. Clin. Endocrinol. Metab. 93 (2), 387–392. 10.1210/jc.2007-1734 18000089 PMC2243226

[B10] Braggio M. Dorelli G. Olivato N. Lamberti V. Valenti M. T. Dalle Carbonare L. (2025). Tailored exercise intervention in metabolic syndrome: Cardiometabolic improvements beyond weight loss and Diet-A prospective observational study. Nutrients 17 (5), 872. 10.3390/nu17050872 40077741 PMC11901541

[B11] Brennan A. M. Standley R. A. Yi F. Carnero E. A. Sparks L. M. Goodpaster B. H. (2020). Individual response variation in the effects of weight loss and exercise on insulin sensitivity and cardiometabolic risk in older adults. Front. Endocrinol. (Lausanne) 11, 632. 10.3389/fendo.2020.00632 33013705 PMC7511700

[B12] Brinkmann C. Weh-Gray O. Brixius K. Bloch W. Predel H. G. Kreutz T. (2019). Effects of exercising before breakfast on the health of T2DM patients-A randomized controlled trial. Scand. J. Med. Sci. Sports 29 (12), 1930–1936. 10.1111/sms.13543 31442336

[B13] Bunprajun T. Henriksen T. I. Scheele C. Pedersen B. K. Green C. J. (2013). Lifelong physical activity prevents aging-associated insulin resistance in human skeletal muscle myotubes *via* increased glucose transporter expression. PLoS One 8 (6), e66628. 10.1371/journal.pone.0066628 23805253 PMC3689670

[B14] Chabi B. Ljubicic V. Menzies K. J. Huang J. H. Saleem A. Hood D. A. (2008). Mitochondrial function and apoptotic susceptibility in aging skeletal muscle. Aging Cell 7 (1), 2–12. 10.1111/j.1474-9726.2007.00347.x 18028258

[B15] Chamari K. Padulo J. (2015). Aerobic' and 'Anaerobic' terms used in exercise physiology: a critical terminology reflection. Sports Med. Open 1 (1), 9. 10.1186/s40798-015-0012-1 27747843 PMC5016084

[B16] Chen N. He X. Feng Y. Ainsworth B. E. Liu Y. (2021). Effects of resistance training in healthy older people with sarcopenia: a systematic review and meta-analysis of randomized controlled trials. Eur. Rev. Aging Phys. Act. 18 (1), 23. 10.1186/s11556-021-00277-7 34763651 PMC8588688

[B17] Chen J. Y. Wu W. T. Lee R. P. Yao T. K. Peng C. H. Chen H. W. (2025). Raloxifene is associated with total knee arthroplasty in postmenopausal women: a comparative cohort study. Life (Basel) 15 (8), 1531. 10.3390/life15101531 41157204 PMC12565639

[B18] Chobanyan-Jürgens K. Scheibe R. J. Potthast A. B. Hein M. Smith A. Freund R. (2019). Influences of hypoxia exercise on whole-body insulin sensitivity and oxidative metabolism in older individuals. J. Clin. Endocrinol. Metab. 104 (11), 5238–5248. 10.1210/jc.2019-00411 30942862

[B19] Christensen R. H. Wedell-Neergaard A. S. Lehrskov L. L. Legaard G. E. Dorph E. Larsen M. K. (2019). Effect of aerobic and resistance exercise on cardiac adipose tissues: secondary analyses from a randomized clinical trial. JAMA Cardiol. 4 (8), 778–787. 10.1001/jamacardio.2019.2074 31268469 PMC6613292

[B20] Čížková T. Štěpán M. Daďová K. Ondrůjová B. Sontáková L. Krauzová E. (2020). Exercise training reduces inflammation of adipose tissue in the elderly: cross-sectional and randomized interventional trial. J. Clin. Endocrinol. Metab. 105 (12), dgaa630. 10.1210/clinem/dgaa630 32902644

[B21] Coates A. M. Joyner M. J. Little J. P. Jones A. M. Gibala M. J. (2023). A perspective on high-intensity interval training for performance and health. Sports Med. 53 (Suppl. 1), 85–96. 10.1007/s40279-023-01938-6 37804419 PMC10721680

[B22] Colleluori G. Viola V. Bathina S. Armamento-Villareal R. Qualls C. Giordano A. (2025). Effect of aerobic or resistance exercise, or both on insulin secretion, ciliary neurotrophic factor, and insulin-like growth factor-1 in dieting older adults with obesity. Clin. Nutr. 51, 50–62. 10.1016/j.clnu.2025.05.016 40527119 PMC12241963

[B23] Consitt L. A. Van Meter J. Newton C. A. Collier D. N. Dar M. S. Wojtaszewski J. F. P. (2013). Impairments in site-specific AS160 phosphorylation and effects of exercise training. Diabetes 62 (10), 3437–3447. 10.2337/db13-0229 23801578 PMC3781473

[B24] Consitt L. A. Dudley C. Saxena G. (2019). Impact of endurance and resistance training on skeletal muscle glucose metabolism in older adults. Nutrients 11 (11), 2636. 10.3390/nu11112636 31684154 PMC6893763

[B25] Cowie C. C. Rust K. F. Ford E. S. Eberhardt M. S. Byrd-Holt D. D. Li C. (2009). Full accounting of diabetes and pre-diabetes in the U.S. population in 1988-1994 and 2005-2006. Diabetes Care 32 (2), 287–294. 10.2337/dc08-1296 19017771 PMC2628695

[B26] Dai C. Hang Y. Shostak A. Poffenberger G. Hart N. Prasad N. (2017). Age-dependent human β cell proliferation induced by glucagon-like peptide 1 and calcineurin signaling. J. Clin. Invest. 127 (10), 3835–3844. 10.1172/JCI91761 28920919 PMC5617654

[B27] Dehghan A. Kardys I. De Maat M. P. Uitterlinden A. G. Sijbrands E. J. G. Bootsma A. H. (2007). Genetic variation, C-reactive protein levels, and incidence of diabetes. Diabetes 56 (3), 872–878. 10.2337/db06-0922 17327459

[B68] De Matos D. G. De Almeida-Neto P. F. Moreira O. C. de Souza R. F. Tinoco Cabral B. G. d. A. Chilibeck P. (2021). Two weekly sessions of high-intensity interval training improve metabolic syndrome and hypertriglyceridemic waist phenotype in older adults: a randomized controlled trial. Metab. Syndr. Relat. Disord. 19(6), 332–339. 10.1089/met.2020.0136 33761288

[B28] Deng Z. Song C. Chen L. Zhang R. Yang L. Zhang P. (2024). Inhibition of CILP2 improves glucose metabolism and mitochondrial dysfunction in sarcopenia *via* the wnt signalling pathway. J. Cachexia Sarcopenia Muscle 15 (6), 2544–2558. 10.1002/jcsm.13597 39385717 PMC11634484

[B29] Distefano G. Standley R. A. Dubé J. J. Carnero E. A. Ritov V. B. Stefanovic-Racic M. (2017). Chronological age does not influence *Ex-vivo* mitochondrial respiration and quality control in skeletal muscle. J. Gerontol. A Biol. Sci. Med. Sci. 72 (4), 535–542. 10.1093/gerona/glw102 27325231 PMC6075361

[B30] Dun Y. Thomas R. J. Smith J. R. Medina-Inojosa J. R. Squires R. W. Bonikowske A. R. (2019). High-intensity interval training improves metabolic syndrome and body composition in outpatient cardiac rehabilitation patients with myocardial infarction. Cardiovasc Diabetol. 18 (1), 104. 10.1186/s12933-019-0907-0 31412869 PMC6694483

[B90] Dos Santos J. M. Benite-Ribeiro S. A. Queiroz G. Duarte J. A. (2012). The effect of age on glucose uptake and GLUT1 and GLUT4 expression in rat skeletal muscle. Cell Biochem. Funct. 30(3), 191–197. 10.1002/cbf.1834 22125125

[B31] Erickson M. L. Malin S. K. Wang Z. Brown J. M. Hazen S. L. Kirwan J. P. (2019). Effects of lifestyle intervention on plasma trimethylamine N-Oxide in Obese adults. Nutrients 11 (1), 179. 10.3390/nu11010179 30654453 PMC6356515

[B32] Fealy C. E. Grevendonk L. Hoeks J. Hesselink M. K. C. (2021). Skeletal muscle mitochondrial network dynamics in metabolic disorders and aging. Trends Mol. Med. 27 (11), 1033–1044. 10.1016/j.molmed.2021.07.013 34417125

[B33] Fragala M. S. Cadore E. L. Dorgo S. Izquierdo M. Kraemer W. J. Peterson M. D. (2019). Resistance training for older adults: position statement from the national strength and conditioning association. J. Strength Cond. Res. 33 (8), 2019–2052. 10.1519/JSC.0000000000003230 31343601

[B34] Gargallo P. Tamayo E. Jiménez-Martínez P. Juesas A. Casaña J. Benitez-Martinez J. C. (2024). Multicomponent and power training with elastic bands improve metabolic and inflammatory parameters, body composition and anthropometry, and physical function in older women with metabolic syndrome: a 20-week randomized, controlled trial. Exp. Gerontol. 185, 112340. 10.1016/j.exger.2023.112340 38061437

[B35] Gaster M. Poulsen P. Handberg A. Schroder H. D. Beck-Nielsen H. (2000). Direct evidence of fiber type-dependent GLUT-4 expression in human skeletal muscle. Am. J. Physiol. Endocrinol. Metab. 278 (5), E910–E916. 10.1152/ajpendo.2000.278.5.E910 10780948

[B36] Ghodrat L. Razeghian Jahromi I. Koushkie J. M. Nemati J. (2022). Effect of performing high-intensity interval training and resistance training on the same day vs. different days in women with type 2 diabetes. Eur. J. Appl. Physiol. 122 (9), 2037–2047. 10.1007/s00421-022-04980-w 35761105

[B37] Gilbertson N. M. Eichner N. Z. M. Francois M. Gaitán J. M. Heiston E. M. Weltman A. (2018). Glucose tolerance is linked to postprandial fuel use independent of exercise dose. Med. Sci. Sports Exerc 50 (10), 2058–2066. 10.1249/MSS.0000000000001667 29762253

[B38] Gomez-Bruton A. Navarrete-Villanueva D. Pérez-Gómez J. Vila-Maldonado S. Gesteiro E. Gusi N. (2020). The effects of age, organized physical activity and sedentarism on fitness in older adults: an 8-Year longitudinal study. Int. J. Environ. Res. Public Health 17 (12), 4312. 10.3390/ijerph17124312 32560257 PMC7345727

[B39] Gouspillou G. Bourdel-Marchasson I. Rouland R. Calmettes G. Biran M. Deschodt-Arsac V. (2014). Mitochondrial energetics is impaired *in vivo* in aged skeletal muscle. Aging Cell 13 (1), 39–48. 10.1111/acel.12147 23919652 PMC4326861

[B40] Guilherme A. Virbasius J. V. Puri V. Czech M. P. (2008). Adipocyte dysfunctions linking obesity to insulin resistance and type 2 diabetes. Nat. Rev. Mol. Cell Biol. 9 (5), 367–377. 10.1038/nrm2391 18401346 PMC2886982

[B41] Ha M. S. Son W. M. (2018). Combined exercise is a modality for improving insulin resistance and aging-related hormone biomarkers in elderly Korean women. Exp. Gerontol. 114, 13–18. 10.1016/j.exger.2018.10.012 30359693

[B42] Heiskanen M. A. Motiani K. K. Mari A. Saunavaara V. Eskelinen J. J. Virtanen K. A. (2018). Exercise training decreases pancreatic fat content and improves beta cell function regardless of baseline glucose tolerance: a randomised controlled trial. Diabetologia 61 (8), 1817–1828. 10.1007/s00125-018-4627-x 29717337 PMC6061150

[B43] Hwang C. L. Yoo J. K. Kim H. K. Hwang M. H. Handberg E. M. Petersen J. W. (2016). Novel all-extremity high-intensity interval training improves aerobic fitness, cardiac function and insulin resistance in healthy older adults. Exp. Gerontol. 82, 112–119. 10.1016/j.exger.2016.06.009 27346646 PMC4975154

[B44] Iannetta D. Inglis E. C. Mattu A. T. Fontana F. Y. Pogliaghi S. Keir D. A. (2020). A critical evaluation of current methods for exercise prescription in women and men. Med. Sci. Sports Exerc 52 (2), 466–473. 10.1249/MSS.0000000000002147 31479001

[B45] Inglis E. C. Iannetta D. Rasica L. Mackie M. Z. Keir D. A. Macinnis M. J. (2024). Heavy-Severe-and Extreme-but not moderate-intensity exercise increase v̇o 2 max and thresholds after 6 wk of training. Med. Sci. Sports Exerc 56 (7), 1307–1316. 10.1249/MSS.0000000000003406 38376995

[B46] Jensen R. C. Christensen L. L. Nielsen J. Schrøder H. D. Kvorning T. Gejl K. (2018). Mitochondria, glycogen, and lipid droplets in skeletal muscle during testosterone treatment and strength training: a randomized, double-blinded, placebo-controlled trial. Andrology 6 (4), 547–555. 10.1111/andr.12492 29656500

[B47] Jeon Y. K. Kim S. S. Kim J. H. Kim H. J. Kim H. J. Park J. J. (2020). Combined aerobic and resistance exercise training reduces circulating apolipoprotein J levels and improves insulin resistance in postmenopausal diabetic women. Diabetes Metab. J. 44 (1), 103–112. 10.4093/dmj.2018.0160 32097999 PMC7043986

[B48] Karstoft K. Clark M. A. Jakobsen I. Knudsen S. H. van Hall G. Pedersen B. K. (2017). Glucose effectiveness, but not insulin sensitivity, is improved after short-term interval training in individuals with type 2 diabetes mellitus: a controlled, randomised, crossover trial. Diabetologia 60 (12), 2432–2442. 10.1007/s00125-017-4406-0 28842722

[B49] Kim J. A. Wei Y. Sowers J. R. (2008). Role of mitochondrial dysfunction in insulin resistance. Circ. Res. 102 (4), 401–414. 10.1161/CIRCRESAHA.107.165472 18309108 PMC2963150

[B50] Kim Y. Triolo M. Hood D. A. (2017). Impact of aging and exercise on mitochondrial quality control in skeletal muscle. Oxid. Med. Cell Longev. 2017, 3165396. 10.1155/2017/3165396 28656072 PMC5471566

[B51] Kim D. I. Lee D. H. Hong S. Jo S. W. Won Y. S. Jeon J. Y. (2018). Six weeks of combined aerobic and resistance exercise using outdoor exercise machines improves fitness, insulin resistance, and chemerin in the Korean elderly: a pilot randomized controlled trial. Arch. Gerontol. Geriatr. 75, 59–64. 10.1016/j.archger.2017.11.006 29190545

[B52] Kim S. W. Jung W. S. Park W. Park H. Y. (2019). Twelve weeks of combined resistance and aerobic exercise improves cardiometabolic biomarkers and enhances red blood cell hemorheological function in Obese older men: a randomized controlled trial. Int. J. Environ. Res. Public Health 16 (24), 5020. 10.3390/ijerph16245020 31835508 PMC6950327

[B53] Ko G. Davidson L. E. Brennan A. M. Lam M. Ross R. (2016). Abdominal adiposity, not cardiorespiratory fitness, mediates the exercise-induced change in insulin sensitivity in older adults. PLoS One 11 (12), e0167734. 10.1371/journal.pone.0167734 27936206 PMC5147957

[B54] Konopka A. R. Laurin J. L. Schoenberg H. M. Reid J. J. Castor W. M. Wolff C. A. (2019). Metformin inhibits mitochondrial adaptations to aerobic exercise training in older adults. Aging Cell 18 (1), e12880. 10.1111/acel.12880 30548390 PMC6351883

[B56] Kuk J. L. Saunders T. J. Davidson L. E. Ross R. (2009). Age-related changes in total and regional fat distribution. Ageing Res. Rev. 8 (4), 339–348. 10.1016/j.arr.2009.06.001 19576300

[B57] Kushner J. A. (2013). The role of aging upon β cell turnover. J. Clin. Invest. 123 (3), 990–995. 10.1172/JCI64095 23454762 PMC3582123

[B58] Lee J. H. Lee J. (2022). Endoplasmic reticulum (ER) stress and its role in pancreatic β-Cell dysfunction and senescence in type 2 diabetes. Int. J. Mol. Sci. 23 (9), 4843. 10.3390/ijms23094843 35563231 PMC9104816

[B59] Li J. Zhang P. Yang L. (2025). Effect of 12-week fitness walking programme on sex hormone levels and risk factors for metabolic syndrome in postmenopausal women: a pilot study. Nutr. Metab. Cardiovasc Dis. 35 (8), 103935. 10.1016/j.numecd.2025.103935 40102114

[B60] Linden M. A. Ross T. T. Beebe D. A. Gorgoglione M. F. Hamilton K. L. Miller B. F. (2019). The combination of exercise training and sodium-glucose cotransporter-2 inhibition improves glucose tolerance and exercise capacity in a rodent model of type 2 diabetes. Metabolism 97, 68–80. 10.1016/j.metabol.2019.05.009 31132381

[B61] Lohne-Seiler H. Kolle E. Anderssen S. A. Hansen B. H. (2016). Musculoskeletal fitness and balance in older individuals (65-85 years) and its association with steps per day: a cross sectional study. BMC Geriatr. 16 (6), 6. 10.1186/s12877-016-0188-3 26755421 PMC4709913

[B62] Low J. L. Marcotte-Chénard A. Tremblay R. Islam H. Falkenhain K. Mampuya W. M. (2025). An acute bout of 4 × 4-min or 10 × 1-min HIIT improves β cell glucose sensitivity in postmenopausal females with type 2 diabetes: a secondary analysis. J. Appl. Physiol. (1985) 138 (1), 311–317. 10.1152/japplphysiol.00777.2024 39694495

[B63] Malin S. K. Syeda U. S. A. (2024). Exercise training independent of intensity lowers plasma bile acids in prediabetes. Med. Sci. Sports Exerc 56 (6), 1009–1017. 10.1249/MSS.0000000000003384 38190376 PMC11096085

[B64] Malin S. K. Gerber R. Chipkin S. R. Braun B. (2012). Independent and combined effects of exercise training and metformin on insulin sensitivity in individuals with prediabetes. Diabetes Care 35 (1), 131–136. 10.2337/dc11-0925 22040838 PMC3241331

[B65] Malin S. K. Frick H. Wisseman W. S. Edwards E. S. Edwards D. A. Emerson S. R. (2023). β-Cell function during a high-fat meal in young versus old adults: role of exercise. Am. J. Physiol. Regul. Integr. Comp. Physiol. 325 (2), R164–R171. 10.1152/ajpregu.00047.2023 37306399 PMC10393366

[B66] Marrs J. C. (2012). Glucose and low-density lipoprotein cholesterol lowering in elderly patients with type 2 diabetes: focus on combination therapy with colesevelam HCl. Drugs Aging 29 (5), e1–e12. 10.2165/11599290-000000000-00000 22530704 PMC3586066

[B67] Mastrandrea C. J. Hajj-Boutros G. Sonjak V. Hedge E. T. Gouspillou G. Hughson R. L. (2025). Exercise attenuates bed rest-induced increases in insulin resistance while α-klotho increases in 55 to 65 year-old women and men. Sci. Rep. 15 (1), 26927. 10.1038/s41598-025-12770-5 40707556 PMC12290009

[B69] Mensberg P. Frandsen C. Carl C. S. Espersen E. Leineweber T. Larsen E. L. (2025). High-intensity interval training improves insulin sensitivity in individuals with prediabetes. Eur. J. Endocrinol. 192 (4), 456–465. 10.1093/ejendo/lvaf004 40235355

[B70] Mi W. Xia Y. Bian Y. (2019). The influence of ICAM1 rs5498 on diabetes mellitus risk: evidence from a meta-analysis. Inflamm. Res. 68 (4), 275–284. 10.1007/s00011-019-01220-4 30798334

[B71] Minamino T. Orimo M. Shimizu I. Kunieda T. Yokoyama M. Ito T. (2009). A crucial role for adipose tissue p53 in the regulation of insulin resistance. Nat. Med. 15 (9), 1082–1087. 10.1038/nm.2014 19718037

[B72] Mingzheng X. You W. (2025). AMPK/mTOR balance during exercise: implications for insulin resistance in aging muscle. Mol. Cell Biochem. 480, 5941–5953. 10.1007/s11010-025-05362-4 40759809

[B73] Mizukami H. Takahashi K. Inaba W. Osonoi S. Kamata K. Tsuboi K. (2014). Age-associated changes of islet endocrine cells and the effects of body mass index in Japanese. J. Diabetes Investig. 5 (1), 38–47. 10.1111/jdi.12118 24843735 PMC4025233

[B74] Moin A. S. M. Cory M. Gurlo T. Saisho Y. Rizza R. A. Butler P. C. (2020). Pancreatic alpha-cell mass across adult human lifespan. Eur. J. Endocrinol. 182 (2), 219–231. 10.1530/EJE-19-0844 31821160 PMC6944979

[B75] Mosalman Haghighi M. Mavros Y. Kay S. Simpson K. A. Baker M. K. Wang Y. (2021). The effect of high-intensity power training on habitual, intervention and total physical activity levels in older adults with type 2 diabetes: secondary outcomes of the GREAT2DO randomized controlled trial. Geriatr. (Basel) 6 (1), 15. 10.3390/geriatrics6010015 33567586 PMC7930974

[B76] Nicklas B. J. Wang X. You T. Lyles M. F. Demons J. Easter L. (2009). Effect of exercise intensity on abdominal fat loss during calorie restriction in overweight and Obese postmenopausal women: a randomized, controlled trial. Am. J. Clin. Nutr. 89 (4), 1043–1052. 10.3945/ajcn.2008.26938 19211823 PMC2667455

[B77] Nishikawa H. Asai A. Fukunishi S. Nishiguchi S. Higuchi K. (2021). Metabolic syndrome and sarcopenia. Nutrients 13 (10), 3519. 10.3390/nu13103519 34684520 PMC8541622

[B78] Normandin E. Chmelo E. Lyles M. F. Marsh A. P. Nicklas B. J. (2017). Effect of resistance training and caloric restriction on the metabolic syndrome. Med. Sci. Sports Exerc 49 (3), 413–419. 10.1249/MSS.0000000000001122 27741216 PMC5315658

[B79] Okamura T. Hashimoto Y. Hamaguchi M. Obora A. Kojima T. Fukui M. (2019). Ectopic fat obesity presents the greatest risk for incident type 2 diabetes: a population-based longitudinal study. Int. J. Obes. (Lond) 43 (1), 139–148. 10.1038/s41366-018-0076-3 29717276

[B80] Oliveira P. F. Gadelha A. B. Gauche R. Paiva F. M. L. Bottaro M. Vianna L. C. (2015). Resistance training improves isokinetic strength and metabolic syndrome-related phenotypes in postmenopausal women. Clin. Interv. Aging 10, 1299–1304. 10.2147/CIA.S87036 26300634 PMC4535561

[B81] Ou M. Y. Zhang H. Tan P. C. Zhou S. B. Li Q. F. (2022). Adipose tissue aging: mechanisms and therapeutic implications. Cell Death Dis. 13 (4), 300. 10.1038/s41419-022-04752-6 35379822 PMC8980023

[B82] Pasini E. Le Douairon Lahaye S. Flati V. Assanelli D. Corsetti G. Speca S. (2012). Effects of treadmill exercise and training frequency on anabolic signaling pathways in the skeletal muscle of aged rats. Exp. Gerontol. 47 (1), 23–28. 10.1016/j.exger.2011.10.003 22015326

[B83] Petersen K. F. Morino K. Alves T. C. Kibbey R. G. Dufour S. Sono S. (2015). Effect of aging on muscle mitochondrial substrate utilization in humans. Proc. Natl. Acad. Sci. U. S. A. 112 (36), 11330–11334. 10.1073/pnas.1514844112 26305973 PMC4568690

[B84] Reaven E. P. Reaven G. M. (1981). Structure and function changes in the endocrine pancreas of aging rats with reference to the modulating effects of exercise and caloric restriction. J. Clin. Invest. 68 (1), 75–84. 10.1172/jci110256 7019247 PMC370774

[B85] Reidy P. T. Lindsay C. C. Mckenzie A. I. Fry C. S. Supiano M. A. Marcus R. L. (2018). Aging-related effects of bed rest followed by eccentric exercise rehabilitation on skeletal muscle macrophages and insulin sensitivity. Exp. Gerontol. 107, 37–49. 10.1016/j.exger.2017.07.001 28705613 PMC5762440

[B86] Ryan A. S. Li G. Mcmillin S. Prior S. J. Blumenthal J. B. Mastella L. (2021). Pathways in skeletal muscle: protein signaling and insulin sensitivity after exercise training and weight loss interventions in middle-aged and older adults. Cells 10 (12), 3490. 10.3390/cells10123490 34943997 PMC8700073

[B87] Sahl R. E. Patsi I. Hansen M. T. Rømer T. Frandsen J. Rasmusen H. K. (2024). Prolonged endurance exercise increases macrophage content and mitochondrial respiration in adipose tissue in trained men. J. Clin. Endocrinol. Metab. 109 (2), e799–e808. 10.1210/clinem/dgad509 37643899

[B88] Saisho Y. Butler A. E. Manesso E. Butler P. C. (2013). Comment on: Saisho et al. β-cell mass and turnover in humans: effects of obesity and aging. Diabetes Care 36 (1), 111–117. 10.2337/dc13-0220 22875233 PMC3526241

[B89] Sano H. Kane S. Sano E. Mîinea C. P. Asara J. M. Lane W. S. (2003). Insulin-stimulated phosphorylation of a rab GTPase-activating protein regulates GLUT4 translocation. J. Biol. Chem. 278 (17), 14599–14602. 10.1074/jbc.C300063200 12637568

[B91] Schwartz R. S. Shuman W. P. Larson V. Cain K. C. Fellingham G. W. Beard J. C. (1991). The effect of intensive endurance exercise training on body fat distribution in young and older men. Metabolism 40 (5), 545–551. 10.1016/0026-0495(91)90239-s 2023542

[B92] Serrano R. Villar M. Gallardo N. Carrascosa J. M. Martinez C. Andrés A. (2009). The effect of aging on insulin signalling pathway is tissue dependent: central role of adipose tissue in the insulin resistance of aging. Mech. Ageing Dev. 130 (3), 189–197. 10.1016/j.mad.2008.11.005 19063913

[B93] Shabkhiz F. Khalafi M. Rosenkranz S. Karimi P. Moghadami K. (2021). Resistance training attenuates circulating FGF-21 and myostatin and improves insulin resistance in elderly men with and without type 2 diabetes mellitus: a randomised controlled clinical trial. Eur. J. Sport Sci. 21 (4), 636–645. 10.1080/17461391.2020.1762755 32345132

[B94] Shiroma E. J. Sesso H. D. Lee I. M. (2012). Physical activity and weight gain prevention in older men. Int. J. Obes. (Lond) 36 (9), 1165–1169. 10.1038/ijo.2011.266 22234277 PMC3326200

[B95] Simpson K. A. Mavros Y. Kay S. Meiklejohn J. de Vos N. Wang Y. (2015). Graded resistance Exercise and Type 2 diabetes in older adults (the GREAT2DO study): methods and baseline cohort characteristics of a randomized controlled trial. Trials 16, 512. 10.1186/s13063-015-1037-y 26554457 PMC4640163

[B96] Son W. M. Park J. J. (2021). Resistance band exercise training prevents the progression of Metabolic syndrome in Obese Postmenopausal women. J. Sports Sci. Med. 20 (2), 291–299. 10.52082/jssm.2021.291 34211322 PMC8219266

[B97] St-Jean-Pelletier F. Pion C. H. Leduc-Gaudet J. P. Sgarioto N. Zovilé I. Barbat-Artigas S. (2017). The impact of ageing, physical activity, and pre-frailty on skeletal muscle phenotype, mitochondrial content, and intramyocellular lipids in men. J. Cachexia Sarcopenia Muscle 8 (2), 213–228. 10.1002/jcsm.12139 27897402 PMC5377417

[B98] Stienstra R. Joosten L. A. Koenen T. van Tits B. van Diepen J. A. van den Berg S. A. A. (2010). The inflammasome-mediated caspase-1 activation controls adipocyte differentiation and insulin sensitivity. Cell Metab. 12 (6), 593–605. 10.1016/j.cmet.2010.11.011 21109192 PMC3683568

[B99] Szoke E. Shrayyef M. Z. Messing S. Woerle H. J. van Haeften T. W. Meyer C. (2008). Effect of aging on glucose homeostasis: accelerated deterioration of beta-cell function in individuals with impaired glucose tolerance. Diabetes Care 31 (3), 539–543. 10.2337/dc07-1443 18083793

[B100] Tan S. Du P. Zhao W. Pang J. Wang J. (2018). Exercise training at maximal fat oxidation intensity for older women with type 2 diabetes. Int. J. Sports Med. 39 (5), 374–381. 10.1055/a-0573-1509 29564847

[B101] Tang F. Wang W. Wang Y. Lee Y. Lou Q. (2024). Moderate resistance training reduces intermuscular adipose tissue and risk factors of atherosclerotic cardiovascular disease for elderly patients with type 2 diabetes. Diabetes Obes. Metab. 26 (8), 3418–3428. 10.1111/dom.15684 38853302

[B102] Tchernof A. Després J. P. (2013). Pathophysiology of human visceral obesity: an update. Physiol. Rev. 93 (1), 359–404. 10.1152/physrev.00033.2011 23303913

[B103] Thyfault J. P. (2008). Setting the stage: possible mechanisms by which acute contraction restores insulin sensitivity in muscle. Am. J. Physiol. Regul. Integr. Comp. Physiol. 294 (4), R1103–R1110. 10.1152/ajpregu.00924.2007 18381969

[B104] Tomeleri C. M. Souza M. F. Burini R. C. Cavaglieri C. R. Ribeiro A. S. Antunes M. (2018). Resistance training reduces metabolic syndrome and inflammatory markers in older women: a randomized controlled trial. J. Diabetes 10 (4), 328–337. 10.1111/1753-0407.12614 29031002

[B55] Von Korn P. Keating S. Mueller S. Haller B. Kraenkel N. Dinges S. (2021). The effect of exercise intensity and volume on Metabolic phenotype in patients with metabolic syndrome: a randomized controlled trial. Metab. Syndr. Relat. Disord. 19(2), 107–114. 10.1089/met.2020.0105 33232639

[B105] Villareal D. T. Banks M. R. Patterson B. W. Polonsky K. S. Klein S. (2008). Weight loss therapy improves pancreatic endocrine function in obese older adults. Obes. (Silver Spring) 16 (6), 1349–1354. 10.1038/oby.2008.226 18388888 PMC2799929

[B106] Viskochil R. Blankenship J. M. Makari-Judson G. Staudenmayer J. Freedson P. S. Hankinson S. E. (2020). Metrics of diabetes risk are only minimally improved by exercise training in postmenopausal breast cancer survivors. J. Clin. Endocrinol. Metab. 105 (5), dgz213. 10.1210/clinem/dgz213 31745553

[B107] Walton R. G. Dungan C. M. Long D. E. Tuggle S. C. Kosmac K. Peck B. D. (2019). Metformin blunts muscle hypertrophy in response to progressive resistance exercise training in older adults: a randomized, double-blind, placebo-controlled, multicenter trial: the MASTERS trial. Aging Cell 18 (6), e13039. 10.1111/acel.13039 31557380 PMC6826125

[B108] Wasserman D. H. (2022). Insulin, muscle glucose uptake, and hexokinase: revisiting the road not taken. Physiol. (Bethesda) 37 (3), 115–127. 10.1152/physiol.00034.2021 34779282 PMC8977147

[B109] Whytock K. L. Goodpaster B. H. (2025). Unraveling skeletal muscle insulin resistance: molecular mechanisms and the restorative role of exercise. Circ. Res. 137 (2), 184–204. 10.1161/CIRCRESAHA.125.325532 40608853 PMC12802857

[B110] Xiao J. Xia Z. Wu Z. Fang M. (2025). Clinical features, treatments, and outcomes of atezolizumab-induced diabetes mellitus in cancer patients. J. Chemother., 1–12. 10.1080/1120009X.2025.2561459 40980946

[B111] Youngren J. F. Barnard R. J. (1985)1995). Effects of acute and chronic exercise on skeletal muscle glucose transport in aged rats. J. Appl. Physiol. 78 (5), 1750–1756. 10.1152/jappl.1995.78.5.1750 7649909

[B112] Zaidi H. Byrkjeland R. Njerve I. U. Åkra S. Solheim S. Arnesen H. (2019). Effects of exercise training on markers of adipose tissue remodeling in patients with coronary artery disease and type 2 diabetes mellitus: sub study of the randomized controlled EXCADI trial. Diabetol. Metab. Syndr. 11, 109. 10.1186/s13098-019-0508-9 31890043 PMC6923919

[B113] Zhang T. Liu Y. Yang Y. Luo J. Hao C. (2025a). The effect and mechanism of regular exercise on improving insulin impedance: based on the perspective of cellular and molecular levels. Int. J. Mol. Sci. 26 (9), 4199. 10.3390/ijms26094199 40362436 PMC12071773

[B114] Zhang Q. Jia Y. Guo Y. Yu X. Wang R. Wang X. (2025b). Chemerin loss-of-function attenuates glucagon-like peptide-1 secretion in exercised obese mice. Diabetes Obes. Metab. 27 (3), 1296–1313. 10.1111/dom.16126 39803714

[B115] Zhou Y. Wu W. Zou Y. Huang W. Lin S. Ye J. (2022). Benefits of different combinations of aerobic and resistance exercise for improving plasma glucose and lipid metabolism and sleep quality among elderly patients with metabolic syndrome: a randomized controlled trial. Endocr. J. 69 (7), 819–830. 10.1507/endocrj.EJ21-0589 35197411

